# Comparison of rapid versus slow maxillary expansion on patient-reported outcome measures in growing patients: a systematic review and meta-analysis

**DOI:** 10.1186/s40510-022-00440-5

**Published:** 2022-12-12

**Authors:** Valentina Rutili, Michele Nieri, Debora Franceschi, Felicita Pierleoni, Veronica Giuntini, Lorenzo Franchi

**Affiliations:** 1 Postgraduate Program in Orthodontics, Department of Experimental and Clinical Medicine, The University of Florence, Florence, Italy; 2 Department of Experimental and Clinical Medicine, The University of Florence, Florence, Italy; 3 Thomas M. Graber Visiting Scholar, Department of Orthodontics and Pediatric Dentistry, School of Dentistry, The University of Michigan, Ann Arbor, USA

**Keywords:** Systematic review, Rapid maxillary expansion, Slow maxillary expansion, Proms, Pain, Meta-analysis

## Abstract

**Background:**

No systematic review and meta-analysis are present in the literature comparing patient-reported outcome measures (PROMs) in rapid maxillary expansion (RME) versus slow maxillary expansion (SME) in growing patients.

**Objective:**

The objective of this systematic review was to compare PROMs in RME versus SME in growing patients.

**Materials and Methods:**

Electronic search in PubMed (MEDLINE), Cochrane Library, Scopus, Embase, Web of Science, and OpenGrey was conducted. Only RCTs were included. Inclusion criteria were: growing patients in the mixed dentition or early permanent dentition, mild-to-moderate maxillary transverse deficiency, dental crowding, treatment with fixed expanders for rapid and slow maxillary expansion. Risk of bias was assessed using RoB 2. GRADE statement was performed. The mean of the differences (MD) and the risk ratio (RR) were used for the aggregation of data. A random effect model was applied.

**Results:**

Two articles with a total of 157 patients were finally included in the systematic review and meta-analysis. One article was at low risk of bias, while one was at risk of bias with some concerns. Pain presence was less, though not statistically significant, in SME patients (RR = 2.02, 95%CI from 0.55 to 7.49, *P* = 0.29, *I*^2^ = 95%, 2 studies, GRADE very low). Pain intensity was significantly lower in SME appliance in the first week of treatment (pooled MD = 0.86 favoring SME, 95%CI from 0.47 to 1.26, *P* < 0.0001, *I*^2^ = 6%, 2 studies, GRADE moderate). There were no significant differences between the two groups in difficulty in speaking, difficulty in swallowing, hypersalivation, difficulty in hygiene, and patient and parent satisfaction.

**Conclusions:**

Pain intensity was significantly lower in SME compared to RME during the first week of treatment. For the following weeks, there were no differences in pain between the two protocols.

## Background

In orthodontic practice, many fixed appliances have been used in growing patients to solve a transverse discrepancy of the maxilla. Fixed jackscrew expander is one of the most used orthodontic appliances to correct this condition [1–3]. Rapid maxillary expansion (RME) is characterized by the application of heavy and intermittent forces in a short time frame that produce an opening of the mid-palatine suture in growing patients. RME can be typically achieved through appliances anchored to teeth or tissues (e.g., Hyrax or Haas) [4]. Slow maxillary expansion (SME) utilizes continuous low-force systems applied over a longer period of time than RME. SME can be produced using different appliances [5–7] (e.g., Quad helix, W arch, expanders incorporating stainless steel or nickel-titanium springs or nickel-titanium wires) or with the same jackscrew expander by using a different activation protocol of the central screw [8–12].

Palatal expanders are effective in expanding the maxilla together with further positive side effects for the patient, such as increasing the size of the airways in the short term [13–15], influencing voice quality [16, 17], and improving hearing [16, 18].

To date, not only the objective benefits of a medical and orthodontic treatment, but also the subjective considerations of the patient are counted, from a patient-centered perspective [19].

In medicine, patient-reported outcomes (PROMs) describe a person’s perception of their health through questionnaires in which patients report on their quality of life, daily functioning, symptoms, and other aspects of their health and well-being [20]. In orthodontics, the evaluation of PROMs is becoming increasingly important, not only because the patient’s psychosocial well-being improves collaboration during therapy [21] but also because the results of orthodontic treatment can be improved if the patient is informed and confident about his or her therapy [22, 23]. It has been proven that RME often produces discomfort or pain especially during the first week of treatment [24–28], particularly in girls [29–32], and it is often associated with an increased age of the child [30, 31]. Other studies showed no differences in gender [25, 27, 33] or age [27, 30, 33] of the patients involved in pain experience after RME treatment. Over time, appliances for SME have been proposed for the correction of maxillary transverse discrepancy. It has been reported that SME produces less tissue resistance in the circum-maxillary structures, better bone formation in the intermaxillary suture [1, 8], and less stress exerted on the midpalatal suture, causing less discomfort for the patient [21].

Rapid and slow expansion protocols have similar efficacy in the treatment of the transverse deficiency of the maxilla [4]. Therefore, given that the two expansion protocols have similar dentoskeletal effect, it is convenient to use an appliance that has minimal negative impact for the patient. For this reason, it is particularly important to evaluate the PROMs when comparing these procedures.

To date, there are few studies evaluating the outcomes reported by patients with SME [6, 21, 24, 26, 34, 35]. No systematic review evaluated PROMs after RME versus SME. Therefore, the aim of this systematic review of randomized controlled trials (RCTs) was to compare PROMs following RME or SME in growing patients.

## Materials and methods

This systematic review was registered (CRD42020221970) at the International Prospective Register of Systematic Reviews (PROSPERO) on December 21, 2020.

### Eligibility criteria

The criteria to select studies were based on the PICOS (Participants, Intervention, Comparison, Outcome, Study) process and are listed in Table 1. Only RCTs were considered.

**Table 1:** Inclusion and exclusion criteria used for study selection (PICOS)

Element	Contents
Participants	Growing patients in the mixed dentition or early permanent dentition, mild-to-moderate maxillary transverse deficiency, dental crowding, treatment with fixed expanders for RME or SME The exclusion criteria were: combined use of facemask treatment, extraction cases, adult treatment, syndromic patients, and surgical cases
Intervention	Conventional fixed jackscrew expanders (either tooth-borne, Hyrax expander, or tooth-tissue-borne, Haas expander) to obtain RME (typically one or two quarters of a turn of the screw per day)
Comparison	Treatment with slow maxillary expansion (SME) achieved with fixed expanders (e.g., jackscrew expander, quad helix, expanders incorporating stainless steel or nickel-titanium springs or nickel-titanium wires)
Outcome	PROMs such as pain, difficulty in speaking, difficulty in swallowing, difficulty in expander hygiene, satisfaction of the patients and the parents
Study design	RCTs (randomized controlled trials)

### Information sources

Electronic search was performed in PubMed (MEDLINE), Cochrane Library, Scopus, Embase, Web of Science, and OpenGrey databases. The survey covered the period from inception to the last access on November 1st, 2021. A manual search was also performed in the references of eligibility studies to find additional relevant articles. No place, language, or publication date restrictions were utilized. Some of the most used registers were included in the databases that were investigated (International Clinical Trials Registry Platform and ClinicalTrials.gov are included in the Cochrane Library database).

### Search strategy

Two search strategies using predefined fields and including a controlled vocabulary (MeSH terms) were applied to identify proper articles. The search strategy is presented in Table 2. The first (1°) was a broad search strategy. The second query string (2°) was developed for the PubMed MEDLINE database search and modified for the other databases respecting the PICOS strategy. After the completion of the search on databases, the results were merged, and all records were imported into a reference management software (EndNote^®^ X9 Thomson Reuters, Philadelphia, PA.). Endnote^®^ software was used to automatically remove duplicate references. After the automatic duplicate’s removal, a manual screening was done to ensure there were no further duplicates.

**Table 2:** Search strategy for electronic databases

Search strategy	Database	Search strategy	Results
1°	PubMed, Cochrane Library, Embase, Scopus, Web of Science, and OpenGrey	Maxillary expansion	PubMed (MEDLINE) (*n* = 4150) Cochrane Library (*n* = 400) Embase (*n* = 3289) Scopus (*n* = 3518) Web of Science (*n* = 4340) OpenGrey (*n* = 5) Total: *n* = 15,702
2°	PubMed	("palatal expansion technique"[MeSH Terms] OR ("palatal"[All Fields] AND "expansion"[All Fields] AND "technique"[All Fields]) OR "palatal expansion technique"[All Fields] OR ("maxillary"[All Fields] AND "expansion"[All Fields]) OR "maxillary expansion"[All Fields]) AND (("child"[MeSH Terms] OR "child"[All Fields]) OR ("adolescent"[MeSH Terms] OR "adolescent"[All Fields]))	PubMed (MEDLINE) (*n* = 2202)
	Cochrane Library, Embase, Scopus, Web of Science, and OpenGrey	["palatal expansion technique" OR (palatal expansion technique)] OR ["maxillary expansion" OR (maxillary expansion)] AND (child OR adolescent)	Cochrane Library (*n* = 388) Embase (*n* = 1841) Scopus (*n* = 2370) Web of Science (*n* = 4000) OpenGrey (*n* = 2) Total: *n* = 10,803
			Total records identified: 26,505

### Selection process

After deleting duplicates, two reviewers (AF and VR) independently analyzed the titles and abstracts of identified records. All articles that did not meet the eligibility criteria were excluded.

The full-text versions of those studies that fulfilled the inclusion criteria, and of those whose content was not clearly based on the information of the title and/or abstract, were acquired.

Then, the same reviewers separately and in double read the full text of the remaining articles applying the eligibility criteria. Any disagreement was resolved through discussion and consensus between the two reviewers, with involvement of a third review author when necessary.

### Data collection process

Data from the articles assessed for eligibility were gather. Information of the included articles comprised the following: study characteristics (authors, year of publication and study design), population characteristics (sample size, gender, and age), clinical evaluation characteristics (type of PROM, type of evaluation scale), characteristics of the results (results presented in relation to the study). If necessary, the authors of the studies were contacted if there were missing elements.

### Data items

The following items of the included studies were collected:

1. Authors and year of the article
2. Study design
3. Sample size, mean age, gender of the subjects, cervical stage
4. Inclusion and exclusion criteria
5. Type of appliance used for expansion
6. Anchorage teeth
7. Activation protocol of the appliance used for expansion
8. Type of PROMs evaluated
9. Type of questionnaire used for PROMs assessment
10. Duration of treatment
11. Follow-up

### Risk of bias assessment

The risk of bias was assessed by two authors (LF and MN) independently and in duplicate. To evaluate the risk of bias of the selected randomized clinical trials, the version 2 of the Cochrane risk-of-bias tool for randomized trials (RoB 2.0) was used [36]. Disagreements between the review authors over the risk of bias were resolved by discussion.

The following biases were analyzed for each included study:

1. Bias arising from the randomization process
2. Bias due to deviations from intended intervention
3. Bias due to missing outcome data
4. Bias in measurement of the outcome
5. Bias in selection of the reported result

Each included study was assigned a global ‘low,’ ‘high,’ or ‘with some concerns’ risk of bias.

### Effect measures and synthesis methods

A narrative synthesis of the findings from the included studies was provided. Clinical, methodological, and statistical heterogeneity was evaluated. If the included studies were sufficiently homogeneous, they were submitted to a quantitative synthesis (meta-analysis) using the Review Manager (RevMan) 5.4.1 software. A random effect model was applied. The mean of the differences (MD) between treatments was reported for the aggregation of continuous data. The outcome effect measure for binary outcomes was expressed as risk ratio. Inverse of variance method and a 95% confidence interval (95% CI) were calculated.

Heterogeneity was assessed through chi-square test (in which a *P* value < 0.1 indicated a statistically significant heterogeneity) and through the inconsistency index (*I*^2^*)*. Values above 50% represented substantial heterogeneity.

The results of the meta-analysis were reported with a forest plot.

If possible, a subgroup analysis by age (children versus adolescents) was planned. A subgroup analysis was also planned based on different activation protocols for the expanders. Another subgroup analysis was planned to include only studies with low risk of bias.

### Reporting bias assessment

Risk of bias of included studies were reported graphically with the risk of bias traffic light plot of ROB2 assessments created using *robvis* [37]. Funnel plot and Egger’s test were proposed to investigate the presence of publication bias if at least 10 studies were included in the meta-analysis.

### Certainty assessment

The certainty of evidence was assessed by the Grading of Recommendations Assessment, Development and Evaluation (GRADE) [38, 39]. The following parameters were assessed by two reviewers (LF and MN): RoB [40], inconsistency (heterogeneity) [41], indirectness [42], imprecision [43] and publication bias [44]. The quality of evidence was classified into four levels: high, moderate, low, and very low.

## Results

### Study selection

**Fig. 1:**
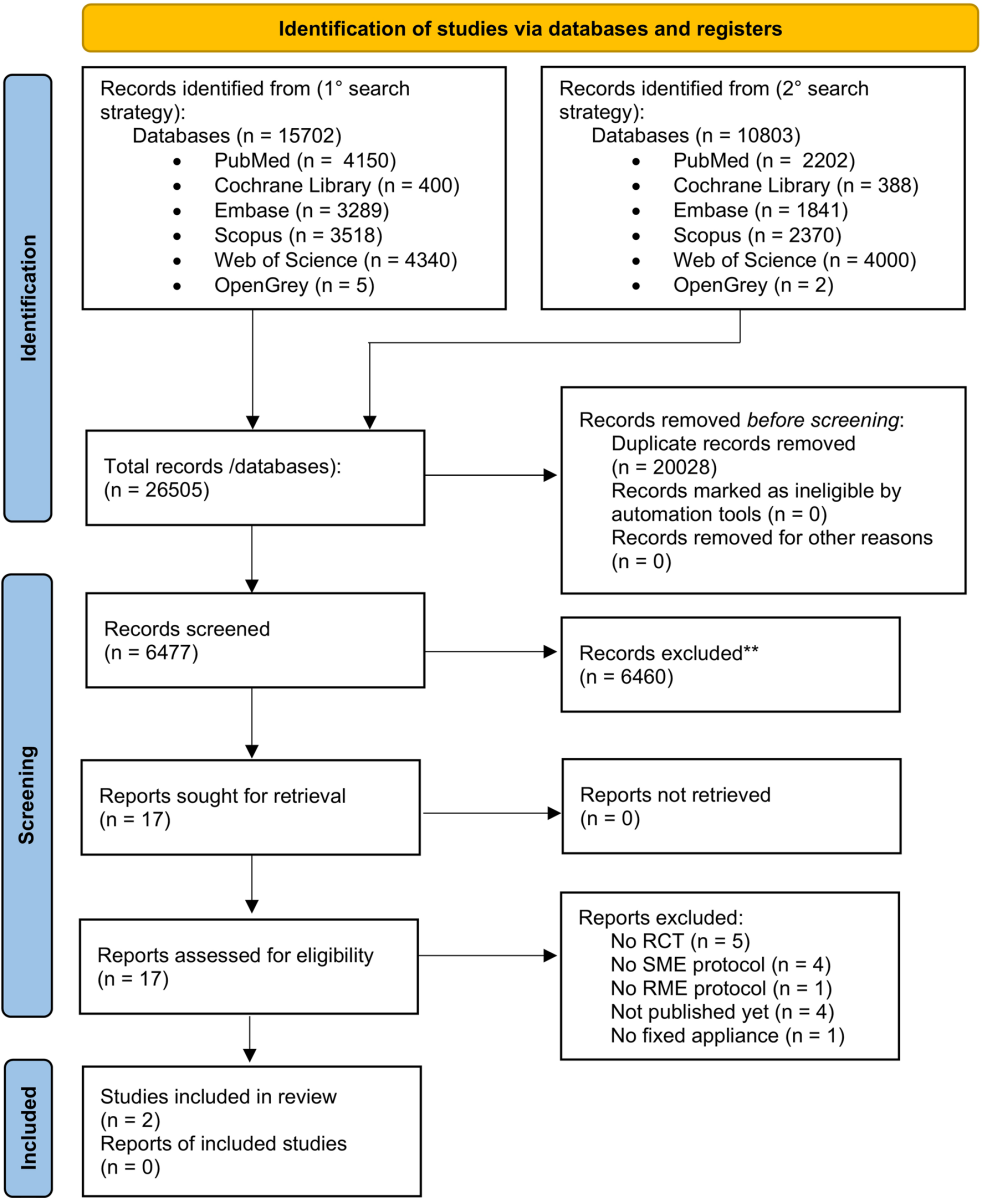
PRISMA 2020 statement flowchart

The full PRISMA 2020 statement flowchart is displayed in Fig. 1.

26,505 total articles were found on the six electronic databases. After removing duplicates, 6477 records remained. After reading the title and abstract, 17 articles remained to be assessed for eligibility. Of these, 15 more were excluded by analyzing the full text. The motivation of their exclusion is reported in Table 3.

**Table 3:** Excluded articles and motivation for exclusion

Study	Motivation for the exclusion
Abed Al Jawad and Alhashimi [21] De Felippe et al. [26] Gecgelen et al. [29] Needleman et al. [33] Serritella et al. [28]	No RCT
Baldini et al. [30] de Araùjo et al. [32] Silveira et al. [50] Halicioglu et al. [27]	No SME protocol
McNally et al. [34]	No RME protocol
ISRCTN [63] NCT [64] NCT [65] NCT [66]	Not published yet
Oshagh et al., 2012 [56]	No fixed appliance

Articles that did not have PROMs as their outcome were excluded by reading the abstract. If it was not clear from the abstract, the full text was retrieved. Many excluded articles were not RCTs. Some articles did not have a group with a slow expansion protocol. A study [27] was excluded because all expanders were activated with a rapid activation protocol, included the memory screw expander which was activated 6 quarter-turns a day.

Two articles were finally included in this systematic review and meta-analysis [24, 35].

### Study characteristics

#### Type of study and location

Characteristics of included articles are presented in Table 4. The two included studies (Ugolini et al. [24]; Nieri et al. [35]) were multicenter RCTs and were performed both within University Departments of Orthodontics. They were both conducted in Italy and published from 2020 to 2021. They both analyzed growing patients who required expansion of the maxilla.

**Table 4:** Characteristics of included studies

Authors of the study	Ugolini et al. [24]	Nieri et al. [35]
Study design	RCT	RCT
Sample size	101 subjects 48 in RME group 53 in SME group	56 subjects 28 in RME group 28 in SME group
Mean age of the subjects	RME: 9.4 years (range 6–13 years) SME: 9.1 years (range 6–13 years)	RME: 8.4 ± 1.0 years SME: 8.0 ± 1.3 years
Sex of the subjects	RME: 26 F; 23 M SME: 28 F; 25 M	RME: 12 F; 16 M SME: 17 F; 11 M
Cervical stage	CS 1—CS 3	RME: 21 subjects in CS 1; 6 subjects in CS 2 SME: 24 subjects in CS 1; 4 subjects in CS 2
Inclusion criteria	Transversal maxillary deficiency (intermolar width < 30 mm, with or without crossbite) Class I or Class II dental malocclusion Cervical vertebral maturation stage 1–3	A posterior transverse interarch discrepancy of at least 3 mm Early or intermediate mixed dentition stage with fully erupted upper and lower first permanent molars Prepubertal (cervical stage 1 or 2)
Exclusion criteria	Previous orthodontic treatment Hypodontia in any quadrant excluding third molars Inadequate oral hygiene craniofacial syndromes, cleft lip or palate	Pubertal or post-pubertal stage of development (CS 3–6) Late deciduous or mixed dentition Agenesis of upper second premolars (assessed on initial panoramic radiograph) Cleft lip and/or palate and craniofacial syndromes Patients unable to be followed for at least 1 year
Type of appliance used for expansion	RME: Hyrax expander SME: Leaf expander	RME: Butterfly expander SME: Leaf expander
Anchorage teeth	Second primary molars	Second primary molars
Activation protocol	RME: two activations at the application of the expander and then two quarter-turns a day, one in the morning and one in the evening (0.40 mm/d) SME: pre-activation in the laboratory to deliver the first 3 mm expansion, and then, reactivation performed in the office by 10 quarter-turns of the screw per month	RME: a quarter of a turn per day SME: pre-activation of the screw causing the first 3 mm of expansion, then reactivation was performed in the office by 10 quarter-turns of the screw per month
Type of PROMs evaluated	Pain (1–7 days of screw activation)—presence, intensity, location, for how many days Jaw function impairment Speaking Salivation (hypersalivation) Swallowing	Pain (in the first 12 weeks)—*primary outcome—*presence, intensity Difficulty in speaking Expander Hygiene Patients’ and parents’ satisfaction
Type of questionnaire used for PROMs assessment	A questionnaire (modified from Feldmann and Bazagani, 2017 [31]) A Wong–Baker Faces Pain Scale with a complementary numeric rating scale from 0 to 10	A questionnaire including all PROMs evaluation VAS and Wong–Baker Faces Pain Scale for pain assessment with a complementary numeric rating scale from 0 to 10
Duration of treatment	Until overcorrection	When the palatal cusps of the upper second deciduous molars approximated the buccal cusps of the lower second deciduous molars
Follow-up	RME: 9 months SME: 9 months	Both expander types were removed 1 year after the start of therapy

#### Characteristics of the participants

In the study by Ugolini et al. [24], the inclusion criteria were a transversal maxillary deficiency, with an intermolar width < 30 mm, with or without crossbite. In the study by Nieri et al. [35], it was not specified if patients had a crossbite, and the inclusion criterion was a posterior interarch discrepancy of at least 3 mm.

In both studies the cervical stage [CS] in cervical vertebral maturation [45] was considered. In one article [35], patients were all prepubertal (CS 1 or CS 2) while in the other article [24] both prepubertal and pubertal patients (CS 1–CS 3) were comprised. Moreover, Ugolini et al. [24] did not specify the dentition stage of patients while in Nieri et al. study [35] patients were in either early or intermediate mixed dentition stage. All studies involved both male and female patients. The age of patients was between 6 and 13 years in one study [24] and between 5.7 and 11.0 years of age in the other study [35].

#### Characteristics of the intervention and comparisons

The included studies [24, 35] compared the outcomes reported by patients after the application of a maxillary expander and after the activation of the appliance. Both studies evaluated the differences of a rapid maxillary expander versus a slow maxillary expander (Leaf expander). The Leaf expander incorporates a Ni–Ti leaf-shaped spring [7] that it is pre-activated to deliver the first 3 mm of expansion. After deactivation, the spring has to be re-activated in office after 2–3 months, by 10 quarter-turns of the screw per month (1 quarter-turn corresponds to 0.1 mm of activation). In Ugolini et al. [24] the RME group was treated by an Hyrax expander which was applied on second primary molars with lingual extensions to the first permanent molars. The expander was activated two times at chairside and then two quarter-turns per day (0.4 mm of expansion per day). In Nieri et al. [35], both the RME group and the Leaf group used a butterfly expander [46] anchored with bands on second primary molars without lingual extensions to the first permanent molars or to the deciduous canines. In the RME group, the protocol of the activation of the screw was a quarter of a turn per day (0.2 mm of expansion per day). In the study by Ugolini et al. [24], the screw of both groups was activated until overcorrection, and then, the expander remained in place for 9 months. In the Nieri et al. [35] study, the expanders were activated until the palatal cusps of the upper second primary molars approximated the buccal cusps of the lower second primary molars. Then, they were left in place and removed after 1 year from the start of treatment.

#### Characteristics of the outcomes

In both studies, the primary outcome was PROMs evaluation. In the study by Ugolini et al. [24], pain in the first week after screw activation was assessed; other outcomes were jaw function impairment such as difficulty in speaking, difficulty in swallowing, and hypersalivation. In Nieri et al. [35], presence of pain was assessed until the 12th week after screw activation. Difficulty in speaking, expander hygiene, and patient and parent satisfaction were also investigated as secondary outcomes.

Pain was considered as a binary outcome (presence/absence) and as a continuous outcome. Intensity of pain assessed through a Visual Analogue Scale (VAS) was included in the meta-analysis until the 4th week of activation of the screw.

### Risk of bias within studies and quality of evidence

**Fig. 2:**
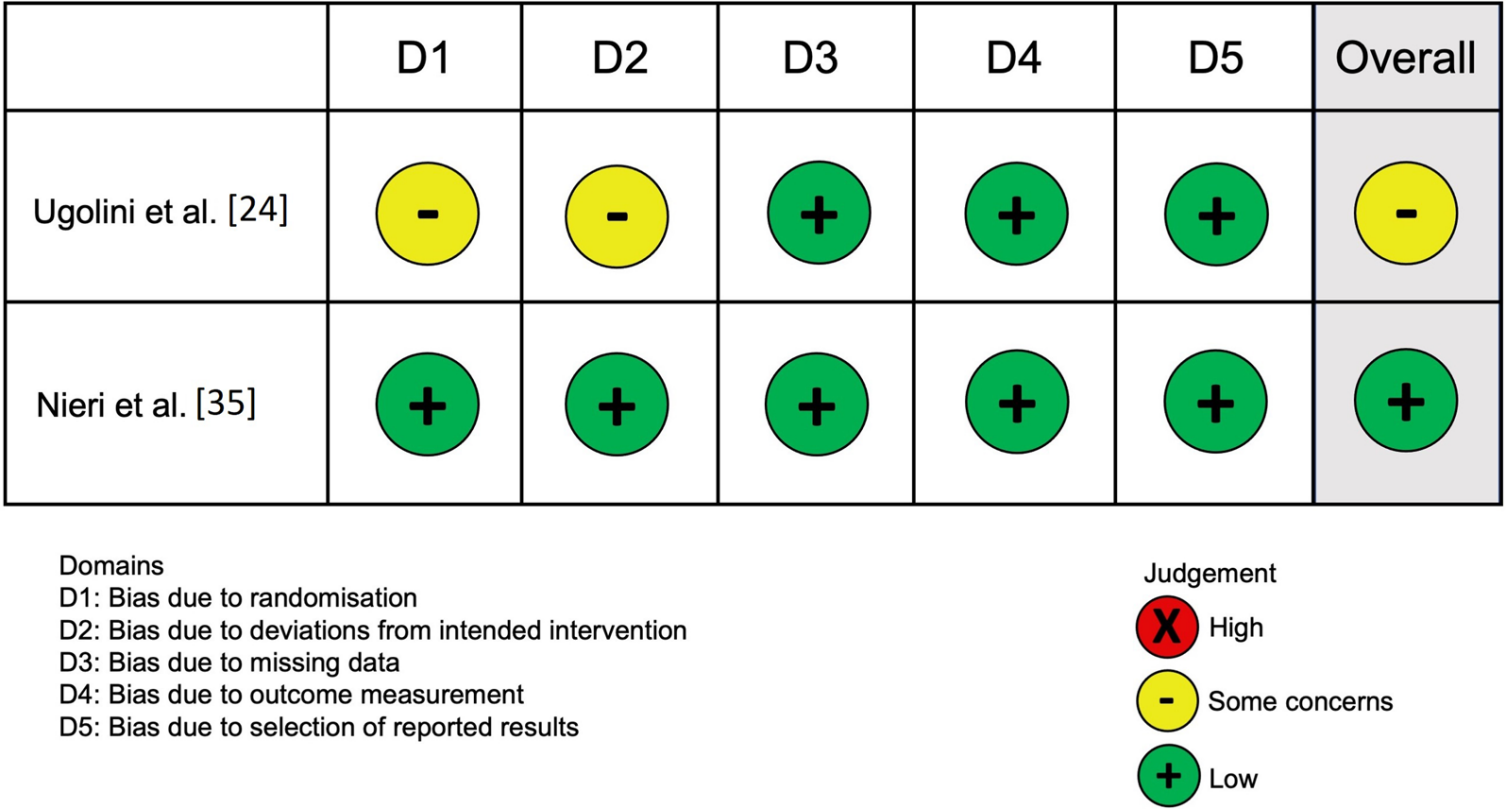
Risk of bias of included studies

The risk of bias of the included RCTs was assessed through the version 2 of the Revised Cochrane risk-of-bias tool for randomized trials (RoB 2), and it is presented graphically in Fig. 2.

#### Overall risk

Ugolini et al. [24], was considered at risk of bias with some concerns.

Nieri et al. [35], was considered at low risk of bias.

Ugolini et al. [24], was considered at risk of bias with some concerns because the allocation concealment was not reported. Moreover, intention-to-treat or modified intention-to-treat analyses were not applied.

#### Quality of evidence

The quality of evidence of included studies was assessed with GRADE. The level of certainty of evidence was moderate for most variables. Summary of Findings Table (SoF) for GRADE statement is presented in Table 5. Indirectness did not affect the level of certainty of evidence because both studies used outcomes that were in agreement with the PICOS questions of the systematic review. Fewer than 10 studies were included in the quantitative synthesis, so it was not possible to assess publication bias. However, the broad search strategy, including the gray literature, diminished the possibility of publication bias.

**Table 5:** Summary of Findings Table (SoF) for GRADE statement of included studies

Outcomes	Mean difference (MD) or Risk ratio (RR) (95% CI)	Number of participants (studies)	Certainty of evidence (GRADE)	Key messages in simple terms
Presence of pain in week 1	2.02 [0.55 to 7.49] (RR)	157 subjects (2 RCTs)	⊕○○○^a,b,c^ Very Low	There is very low evidence that SME appliance results in little or no difference in presence of pain compared to RME appliance (during the first week of screw activation)
VAS Pain week 1	0.86 [0.47 to 1.26] (MD)	157 subjects (2 RCTs)	⊕⊕⊕○^a^ Moderate	The use of an SME appliance probably is associated with less pain intensity during the first week compared to RME appliance
VAS Pain week 2	0.70 [− 0.24 to 1.64] (MD)	56 subjects (1 RCTs)	⊕⊕⊕○^c^ Moderate	There is probably no difference between RME and SME treatment in pain intensity during the second week
VAS Pain week 3	0.20 [− 0.25 to 0.65] (MD)	56 subjects (1 RCTs)	⊕⊕⊕○^c^ Moderate	There is probably no difference between RME and SME treatment in pain intensity during the third week
VAS Pain week 4	0.30 [− 0.17 to 0.77] (MD)	56 subjects (1 RCTs)	⊕⊕⊕ ○^c^ Moderate	There is probably no difference between RME and SME treatment in pain intensity during the fourth week
Presence of difficulty in speaking week 1	0.95 [0.85 to 1.06] (RR)	157 subjects (2 RCTs)	⊕⊕⊕ ○^a^ Moderate	There is probably no difference in difficulty of speaking between RME and SME groups
Presence of difficulty in swallowing week 1	0.93 [0.78 to 1.12] (RR)	101 subjects (1 RCTs)	⊕⊕○○^a,c^ Low	There is low evidence of no difference in difficulty of speaking between RME and SME groups
Hypersalivation week 1	1.03 [0.84 to 1.24] (RR)	101 subjects (1 RCTs)	⊕⊕○○^a,c^ Low	There is low evidence of the absence of difference in hypersalivation in the two groups during the first week of treatment
VAS difficulty in hygiene week 1	0.30 [− 1.14 to 1.74] (MD)	56 subjects (1 RCTs)	⊕⊕⊕○^c^ Moderate	There is probably no difference between the two groups in difficulty of expander hygiene
Patient satisfaction	0.00 [− 0.92 to 0.92] (MD)	56 subjects (1 RCTs)	⊕⊕⊕○^c^ Moderate	The use of RME and SME appliance probably do not differ in patient satisfaction
Parent satisfaction	0.10 [− 0.56 to 0.76] (MD)	56 subjects (1 RCTs)	⊕⊕⊕○^c^ Moderate	The use of RME and SME appliance probably do not differ in parent satisfaction

Participants: growing patients with constricted maxilla

Intervention: treatment with conventional fixed jackscrew expanders to obtain RME

Comparison: treatment with slow maxillary expansion (SME) achieved with fixed expanders

Outcome: patient-reported outcome measures (PROMs)

Study: RCTs

95% CI: 95% confidence interval

GRADE: Evidence grades. Grading of Recommendations Assessment, Development and Evaluation

Domains that lower the level of evidence:

^a^RoB (result from a study with risk of bias with some concerns)

^b^Inconsistency: high statistical heterogeneity across studies

^c^Imprecision: wide confidence interval or only one study

### Results of individual studies and data synthesis

Four binary outcomes (presence of pain in the first week; presence of difficulty of speaking in the first week; presence of difficulty of swallowing in the first week; and hypersalivation in the first week) and 7 continuous outcomes (VAS pain in the first, second, third, and four weeks; VAS difficulty in hygiene in the first week; patient satisfaction; and parent satisfaction) were included in the meta-analysis.

A subgroup analysis by age (children versus adolescents) was not performed due to the similar age of patients of the two studies. Subgroup analysis based on the different activation protocols of the expanders was not performed because only two studies with different activation protocols were included.

Only one study [35] was at low risk of bias and therefore a subgroup analysis was not performed.

#### Summary of the data

The summary results of the data are expressed in Table 6.

**Table 6:** Comparison between patient-related outcomes produced by RME versus SME in included studies in the systematic review

Study	RME		SME	
	Events/mean	Total/SD	Events/mean	Total/SD
*Ugolini et al. *[24] sample size: 101 patients (48 in RME group; 53 in SME group)				
1. Pain presence in the first week	43 (Events)	48 (Total)	13 (Events)	53 (Total)
2. Pain (VAS scale) 1 week	1.6 (Mean)	1 (SD)	0.8 (Mean)	0.7 (SD)
3. Presence of difficulty in speaking in 1 week	42 (Events)	48 (Total)	49 (Events)	53 (Total)
4. Presence of difficulty in swallowing in 1 week	38 (Events)	48 (Total)	45 (Events)	53 (Total)
5. Hypersalivation	39 (Events)	48 (Total)	42 (Events)	53 (Total)

*Nieri et al. *[35] sample size: 56 patients (28 in RME group; 28 in SME group)				
1. Pain presence in 1 week	23 (Events)	28 (Total)	20 (Events)	28 (Total)
2. Pain (VAS scale) in 1 week	3.7 (Mean)	2.6 (SD)	2.2 (Mean)	2.3 (SD)
3. Pain (VAS scale) in 2 week	1.7 (Mean)	1.7 (SD)	1.0 (Mean)	1.9 (SD)
4. Pain (VAS scale) in 3 week	0.5 (Mean)	1.0 (SD)	0.3 (Mean)	0.7 (SD)
5. Pain (VAS scale) in 4 week	0.4 (Mean)	1.2 (SD)	0.1 (Mean)	0.4 (SD)
6. Presence of difficulty in speaking in 1 week	23 (Events)	28 (Total)	24 (Events)	28 (Total)
7. Difficulty in hygiene in 1 week	3.0 (Mean)	2.8 (SD)	2.7 (Mean)	2.7 (SD)
8. Patients’ satisfaction	8.8 (Mean)	1.6 (SD)	8.8 (Mean)	1.9 (SD)
9. Parent’s satisfaction	9.2 (Mean)	1.2 (SD)	9.1 (Mean)	1.3 (SD)

#### Pain

In both studies, pain was assessed as a binary outcome (presence/absence) and as a continuous outcome (intensity of pain through a numerical and visual rating scale).

**Fig. 3:**
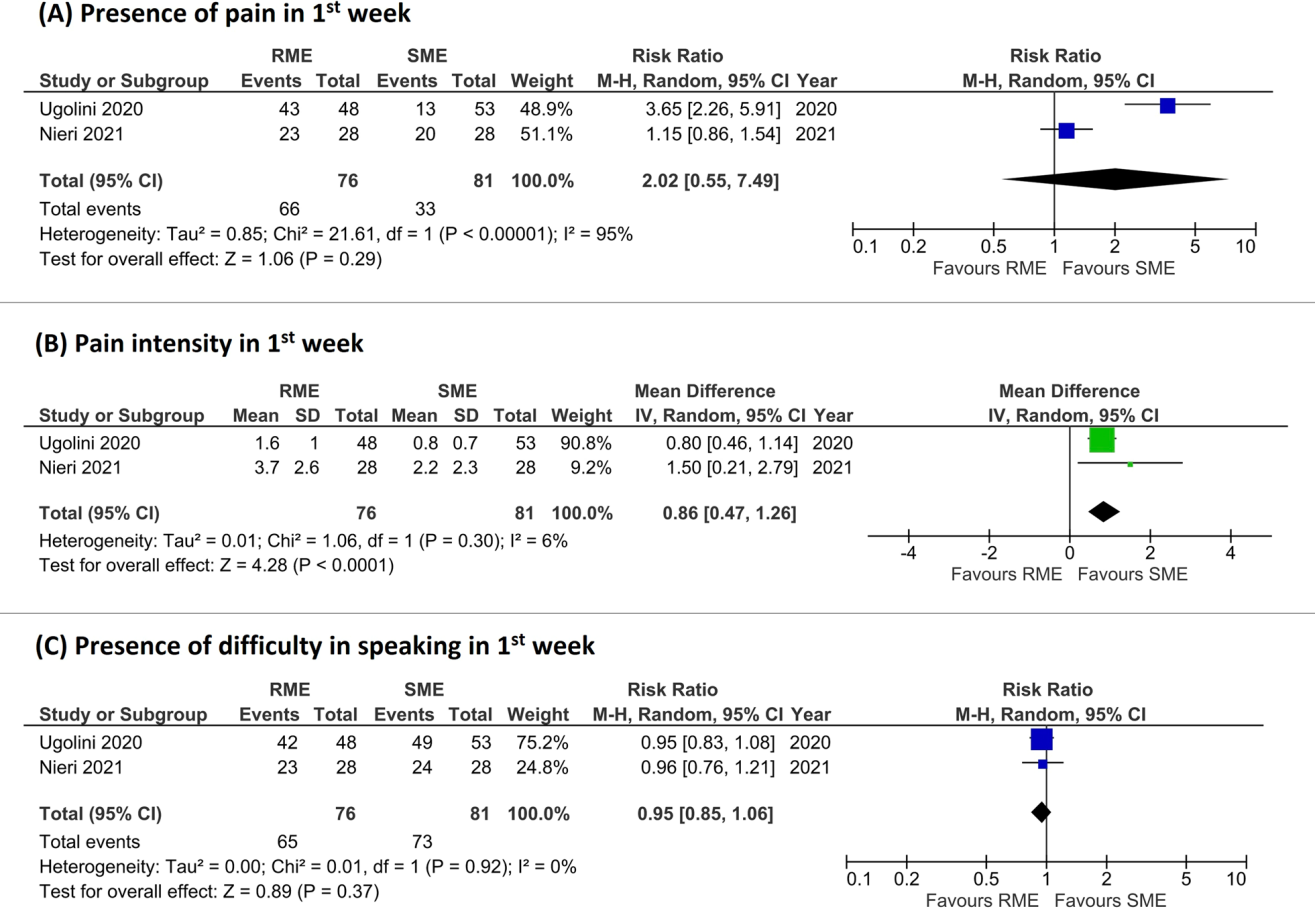
Forest plots of the outcomes included in the meta-analysis (**A**, presence of pain in 1st week; **B**, pain intensity in 1st week; **C**, presence of difficulty in speaking in 1st week)

In one study [24], the visual Wong–Baker scale was employed plus a numeric rating scale. Similarly, in the other study [35] a VAS plus the visual Wong–Baker scale was used. In the study conducted by Ugolini et al. [24], pain was assessed only in the first week of activation of the screw. In Nieri et al. [35], pain was evaluated in the 12 weeks from the screw activation. For the intensity of pain, the first week of treatment for one study [24] and only the first 4 weeks of treatment of the other study [35] were included in the meta-analysis. For the presence of pain in RME and SME, pain was perceived by less patients treated with SME (pooled RR = 2.02 favoring SME, 95% CI from 0.55 to 7.49, *P* = 0.29, 2 studies, GRADE very low). Heterogeneity was significantly high (*I*^2^ = 95%) (Fig. 3A).

**Fig. 4:**
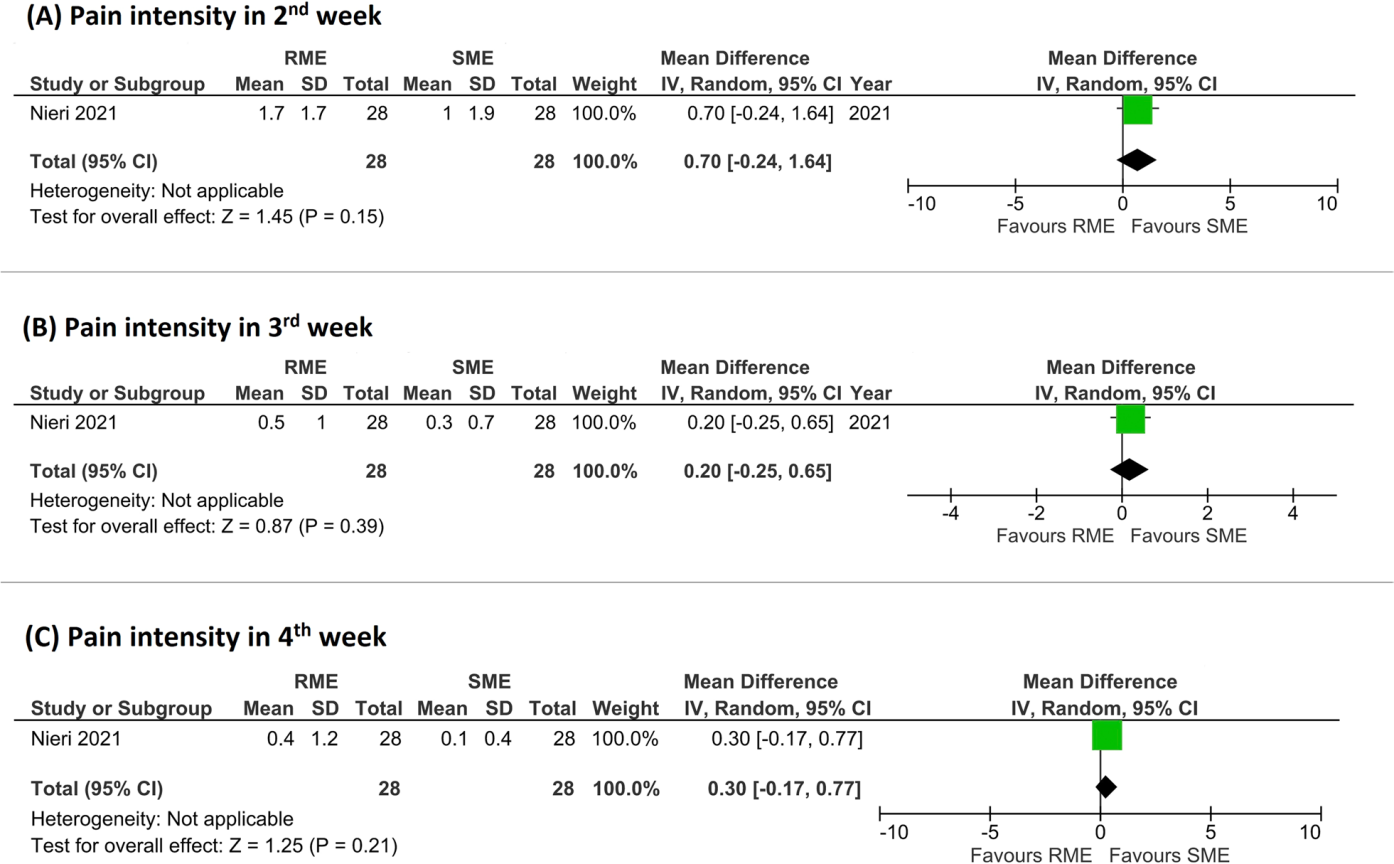
Forest plots of the outcomes included in the meta-analysis (**A**, pain intensity in 2nd week; **B**, pain intensity in 3rd week; **C**, pain intensity in 4th week)

Intensity of pain in the first week was significantly less in patients treated with SME (pooled MD = 0.86 favoring SME, 95% CI from 0.47 to 1.26, *P* < 0.0001, *I*^2^ = 6%, 2 studies, GRADE moderate) (Fig. 3B). In the second week, pain intensity was decreased in both RME and SME, with no significant differences between RME and SME (pooled MD = 0.70 favoring SME, 95% CI from − 0.24 to 1.64, *P* = 0.15, 1 study, GRADE moderate) (Fig. 4A).

In 3rd week, pain intensity was decreased in the two groups, with no significant differences between RME and SME (pooled MD = 0.20 favoring SME, 95% CI from − 0.25 to 0.65, *P* = 0.39, 1 study, GRADE moderate) (Fig. 4B).

In forth week, pain intensity decreased in both RME and SME. There were no significant differences between RME and SME (pooled MD = 0.30 favoring SME, 95% CI from − 0.17 to 0.77, *P* = 0.21, 1 study, GRADE moderate) (Fig. 4C).

#### Difficulty in speaking

Both studies evaluated difficulty in speaking after the first week of beginning of treatment as a binary variable. In both RME and SME difficulty in speaking was highly prevalent (85–90%). In the meta-analysis no significant differences were found between RME and SME (pooled RR = 0.95 favoring RME, 95% CI from 0.85 to 1.06, *P* = 0.37, *I*^2^ = 0%, 2 studies, GRADE moderate) (Fig. 3C).

#### Difficulty in swallowing

**Fig. 5:**
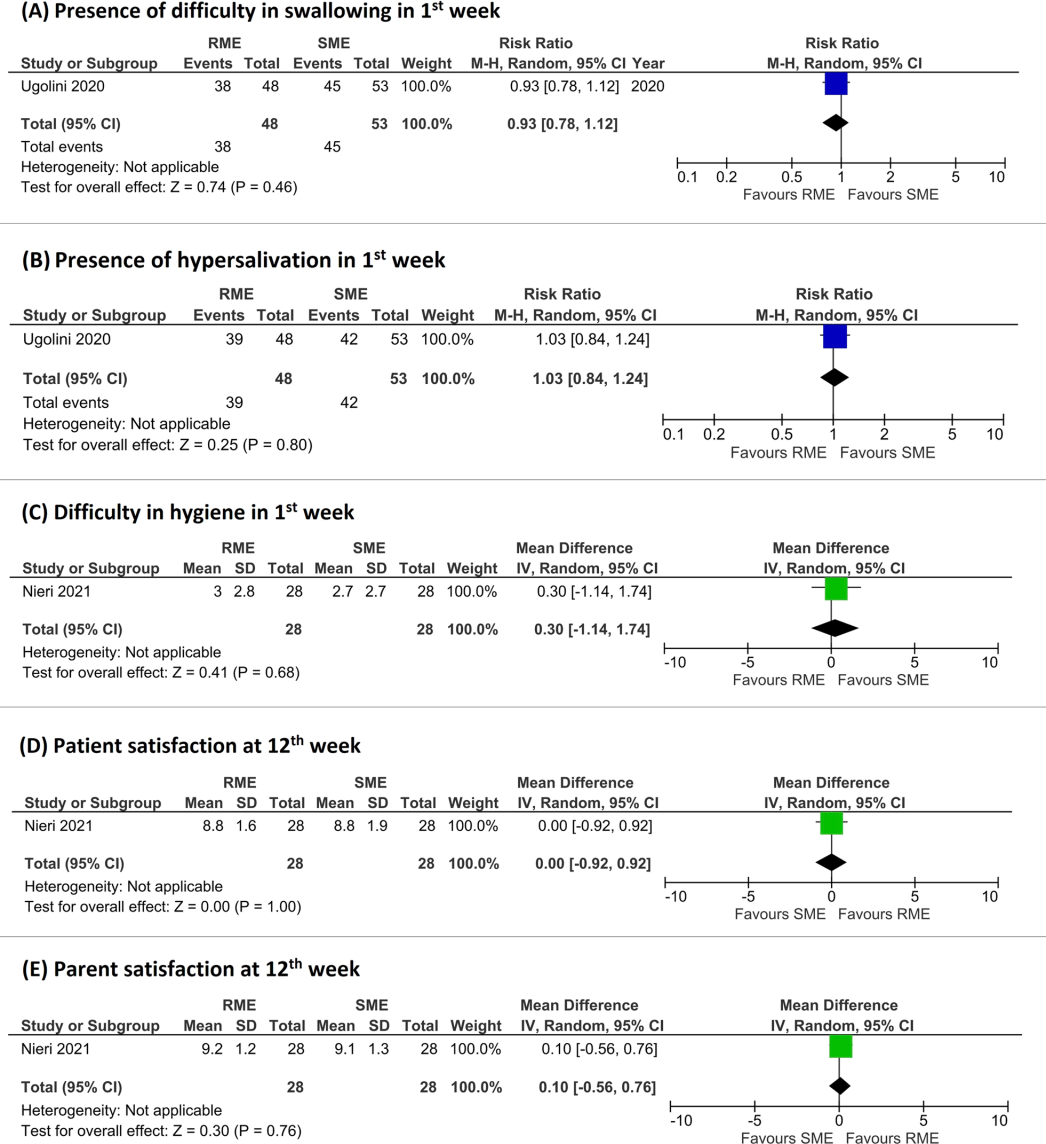
Forest plots of the outcomes included in the meta-analysis (**A**, presence of difficulty in swallowing in 1st week; **B**, presence of hypersalivation in 1st week; **C**, difficulty in hygiene in 1st week; **D**, patient satisfaction at 12th week;** E**, parent satisfaction at 12th week)

Only one study [24] reported data for this PROM as a binary variable after the first week of activation of the screw. Difficulty in swallowing was very frequent in the first week, in both RME and SME (about 80%). The forest plot revealed no significant differences between the two treatment modalities (pooled RR = 0.93 favoring RME, 95% CI from 0.78 to 1.12, *P* = 0.46, 1 study, GRADE low) (Fig. 5A).

#### Hypersalivation

Presence of salivation (hypersalivation) was reported only by Ugolini et al. [24]. Hypersalivation was present in more than 80% on the subjects in the first week of treatment in both RME and SME. The forest plot showed that there were no differences between RME and SME (pooled RR = 1.03 favoring SME, 95% CI from 0.84 to 1.24, *P* = 0.80, 1 study, GRADE low) (Fig. 5B).

#### Difficulty in hygiene

Nieri et al. [35] reported the grading of difficulty in hygiene in the 12th weeks of treatment through the VAS. Difficulty in hygiene was relatively mild in the first week (2.7 in SME group and 3.0 in RME group). No statistically significant differences were reported between RME and SME in the first week (pooled MD = 0.30 favoring SME, 95% CI from − 1.14 to 1.74, *P* = 0.68, 1 study, GRADE moderate) (Fig. 5C).

#### Patient satisfaction

The forest plot demonstrated the absence of difference in patient satisfaction between RME and SME (pooled MD = 0.00, 95% CI from − 0.92 to 0.92, *P* = 1.0, 1 study, GRADE moderate) (Fig. 5D).

#### Parent satisfaction

Only the study by Nieri et al. [35] took this parameter into investigation. The forest plot revealed that no differences existed between RME and SME (pooled MD = 0.10 favoring RME, 95% CI from − 0.56 to 0.76, *P* = 0.76, 1 study, GRADE moderate) (Fig. 5E).

## Discussion

### Summary of evidence

This systematic review aimed to compare patient-reported outcomes after RME versus SME. In orthodontics, patient satisfaction is one of the most generally measured PROMs, especially in adults [23, 47]. As for the expansion of the maxilla in growing patients, the most investigated and most reported PROMs were pain and speech [26, 48].

As for pain, in the first week there was no significant difference for the presence of pain while there was a significant difference for the intensity of pain. With reference to the presence of pain during the first week of activation of the screw, from this systematic review arose that pain was perceived more in the RME group, more than twice than in the SME group (86.8% in RME group vs. 40.7% in SME group) (Fig. 3A). The RR of 2.02, however, was not statistically significant because of the high heterogeneity between the 2 studies. It should be noted that the RR reported by Ugolini et al. [24] was 3.65, while it was only 1.15 in Nieri et al. [35] study. This difference in RR in perceived pain during the first week could have been influenced by the fact that in Ugolini et al. [24] the expansion screw in the RME group was activated 2 times per day versus 1 time per day in Nieri et al. [35] Moreover, in Ugolini et al. [24] the child’s pain response was measured 5 min after each turn while in Nieri et al. [35] pain was recorded after 1 week of treatment with RME. Other factors that could have influenced the difference in RR in perceived pain during the first week between the 2 studies, were the small difference in age of the participants of the two studies and the intensity of the forces generated by the Ni–Ti springs of the Leaf expander in the SME group. In facts, in Ugolini et al. [24] the Leaf expander generated 450 g of force while Nieri et al. [35] used a Leaf expander that produced 900 g of force.

On the contrary, intensity of pain in the first week was significantly less in SME group compared to RME group, with a difference of almost 1 on VAS (Fig. 3B). One study demonstrated a minimum clinically significant difference of 1 on VAS in children [49]. Therefore, during the 1st week the difference in intensity of pain between the 2 groups was nearly clinically significant. In the following weeks, pain decreased progressively, especially in RME group, from about 2.6 in the first week to 0.4 in the fourth week (Fig. 4). Over time, pain in SME group decreased as well, about 1 point on VAS. Pain was assessed every day in the first week only in the study by Ugolini et al. [24] in which pain was perceived more in both groups especially during the first 4 days. This is in accordance with previous studies in particular during RME treatment [27, 29, 31, 32, 50]. In one study [35], pain was evaluated until 12th week; it decreased quickly after 3rd week, and from the 5th week to the 12th week it was almost nil, around 0.1 on VAS. These findings support previous non-randomized studies [21, 26] that compared RME and SME appliances, in which pain was perceived mostly during the first week of treatment, in high percentage in RME groups (93.9% [26] and > 90% in the first 2 days [21]), and then decreased until the beginning of adaptation after the third day [21] or at the end of the first week [26]. Pain was perceived more during the first phase of RME activation due to an inflammatory-like reaction of a highly cellular disorganized connective tissue [51, 52]. As expansion continued, less pain was perceived due to less distraction of the midpalatal tissues followed with each progressive turn of the screw [33].

For the presence of difficulty in speaking during the first week, there was no difference between the two groups. This outcome was measured in both studies included. Difficulty in speaking was highly represented in both treatments in the first week (about 85.5% in RME group and 90% in SME group). During time, difficulty in speaking decreased progressively although it did not disappear completely even at the 12th week, reaching values of 0.2–0.4 on the VAS (Fig. 3C) [35].

A similar trend is displayed for the presence of difficulty in swallowing during the first week, and there was a high prevalence of this condition among patients in both groups (about 79% in RME group and about 85% in SME group). This outcome was measured only in one study [24]. Also for this outcome there were no significant differences between the two groups.

These findings support previous studies in which functional jaw impairment such as difficulty in speaking and difficulty in swallowing were present in expansion treatment mostly during the first week after the cementation of the appliance [21, 26]. After a short period of time, the discomfort in speaking is minimized due to a functional adaptation of the muscles and joints [26]. In children with a narrow palate, the application of the expander causes problems in distorting the /s/ sound because the expander diminishes the tongue’s functional place [53].

It must be stressed that the size and encumbrance of the appliance can be a great impediment to functional movements. Problems in speaking or swallowing are caused by the presence of a foreign body/appliance in the oral cavity [54], especially when considering fixed appliances that cannot be removed by the patient during the day, compared with removable appliance [55]. In this systematic review all included RCTs considered two types of expanders similar in their size and encumbrance in the palatal vault. In a previous non-randomized study conducted by Abed and Alhashimi [21] difficulty in swallowing was extremely different in the two RME (Hyrax) and SME (Quad Helix) appliances. The use of a Quad Helix for SME treatment increased the intraoral space and tongue movements were less restricted for food bolus movements during the second stage of swallowing [21].

The present systematic review showed that there was no difference in salivation (hypersalivation) between RME and SME. This parameter was investigated only in one study [24]. Hypersalivation was high in the first week of treatment (more than 80% in both groups) (Fig. 5B). The presence and quantity of saliva during a treatment with a palatal expander is poorly documented in the literature. Orthodontic palatal appliances cause a salivary overflow especially during the first days as the bulk of the appliance may interfere with the mobility of the tongue and cheeks [56].

Difficulty in hygiene was assessed through the VAS only in one study [35]. This outcome was maximum during the first week of treatment and decreased along with time, without ever reaching 0 point on VAS even at the 12th week [35]. According to the meta-analysis, in the first week of treatment there were no differences between the two groups (Fig. 5C). This result is plausible as the two types of expanders (Hyrax/Butterfly expander and Leaf expander) are very similar in shape and cleaning capacity.

Patient and parent satisfaction were investigated with a VAS only in the study by Nieri et al. [35] in which 0 meant ‘maximum dissatisfaction’ and 10 ‘maximum satisfaction’ with the result assessed at the end of the study. High levels of patient and parent satisfaction were present with no differences between the two groups (Fig. 5D and E).

Usually, patient satisfaction varies largely from a strong disposition to undergo orthodontic treatment (especially in adults) to a complete indifference to treatment, especially in children. Moreover, some children and adolescents had orthodontic treatment because of their parents’ desires [47]. For patients, pain and discomfort during treatment strongly affected treatment satisfaction [23, 57].

### Limitations

PROMs are subjective assessments that are difficult to standardize especially in children [58, 59]. Despite this, numerical rating scales and visual and color analog scales have proved to be understood and properly utilized in growing patients [60, 61]. Pain report in children through self-reports, however, must be interpreted cautiously [33]. In one study included in this systematic review, some children were quite young (< 6 years of age) [35].

A limitation of this study was that review authors were the same as for an article [35] included in this systematic review and meta-analysis.

Another limitation was that few RCTs in the literature compared RME and SME appliances by using PROMs. Of these RCTs, SME was performed only with the Leaf expander and there is lack of information about other types of SME. Additionally, one outcome variable (presence of pain in 1st week) showed high heterogeneity. There were some treatment differences in the two included studies and different modalities of screw activation, although the design of the expanders was quite similar. There were also differences regarding the characteristics of patients included in the two studies. All patients in any case needed maxillary expansion due to transverse discrepancy between the dental arches. More RCTs are needed in orthodontics that include an evaluation of PROMs.

## Conclusions

In growing patients, the application of SME reduced pain intensity compared to RME during the first week of treatment. There were no differences in the first week of treatment for difficulty of speaking, difficulty in swallowing, hypersalivation, difficulty in hygiene, and patient and parent satisfaction between RME and SME appliances. There were no statistically significant differences in pain between the two protocols for all following weeks.

## Data Availability

The data underlying this article will be shared to the corresponding author after reasonable request.
